# Exploring nasopharyngeal microbiota profile in children affected by SARS-CoV-2 infection

**DOI:** 10.1128/spectrum.03009-23

**Published:** 2024-01-30

**Authors:** L. Romani, F. Del Chierico, S. Pane, M. V. Ristori, I. Pirona, V. Guarrasi, N. Cotugno, S. Bernardi, L. Lancella, C. F. Perno, P. Rossi, A. Villani, A. Campana, P. Palma, L. Putignani, the CACTUS Study Team, Francesca Calò Carducci

**Affiliations:** 1 Infectious Disease Unit, Bambino Gesù Children’s Hospital, IRCCS, Rome, Italy; 2 Research Unit of Human Microbiome, Bambino Gesù Children's Hospital, IRCCS, Rome, Italy; 3 Unit of Microbiomics, Bambino Gesù Children’s Hospital, IRCCS, Rome, Italy; 4 GenomeUp SRL, Viale Pasteur, Rome, Italy; 5 Research Unit of Congenital and Perinatal Infections, Bambino Gesù Children’s Hospital, IRCCS, Rome, Italy; 6 Department of Systems Medicine, University of Rome ‘‘Tor Vergata’’, Rome, Italy; 7 Unit of Microbiology and Diagnostic Immunology, Bambino Gesù Children's Hospital, IRCCS, Rome, Italy; 8 Academic Department of Pediatrics, Bambino Gesù Children’s Hospital, IRCCS, Rome, Italy; 9 Pediatric Emergency Department and General Pediatrics, Bambino Gesù Children's Hospital Bambino Gesù, IRCCS, Rome, Italy; 10 Department of Pediatrics, Bambino Gesù Children's Hospital, IRCCS, Rome, Italy; 11 Unit of Microbiomics and Research Unit of Human Microbiome, Bambino Gesù Children's Hospital, IRCCS, Rome, Italy; BGI Group, Yantian, Shenzhen, China

**Keywords:** COVID-19, nasopharyngeal microbiota, children, SARS-CoV-2, respiratory tract

## Abstract

The relationship between COVID-19 and nasopharyngeal (NP) microbiota has been investigated mainly in the adult population. We explored the NP profile of children affected by COVID-19, compared to healthy controls (CTRLs). NP swabs of children with COVID-19, collected between March and September 2020, were investigated at the admission (*T*_0_), 72 h to 7 days (*T*_1_), and at the discharge (*T*_2_) of the patients. NP microbiota was analyzed by 16S rRNA targeted-metagenomics. Data from sequencing were investigated by QIIME 2.0 and PICRUSt 2. Multiple machine learning (ML) models were exploited to classify patients compared to CTRLs. The NP microbiota of COVID-19 patients (*N* = 71) was characterized by reduction of α-diversity compared to CTRLs (*N* = 59). The NP microbiota of COVID-19 cohort appeared significantly enriched in *Streptococcus*, *Haemophilus*, *Staphylococcus*, *Veillonella*, *Enterococcus*, *Neisseria*, *Moraxella*, Enterobacteriaceae, *Gemella*, *Bacillus*, and reduced in *Faecalibacterium*, *Akkermansia*, *Blautia*, *Bifidobacterium*, *Ruminococcus*, and *Bacteroides,* compared to CTRLs (FDR < 0.001). Exploiting ML models, *Enterococcus, Pseudomonas, Streptococcus, Capnocytopagha, Tepidiphilus, Porphyromonas, Staphylococcus*, and *Veillonella* resulted as NP microbiota biomarkers, in COVID-19 patients. No statistically significant differences were found comparing the NP microbiota profile of COVID-19 patients during the time-points or grouping patients on the basis of high, medium, and low viral load (VL). This evidence provides specific pathobiont signatures of the NP microbiota in pediatric COVID-19 patients, and the reduction of anaerobic protective commensals. Our data suggest that the NP microbiota may have a specific disease-related signature since infection onset without changes during disease progression, regardless of the SARS-CoV-2 VL.

**IMPORTANCE:**

Since the beginning of pandemic, we know that children are less susceptible to severe COVID-19 disease. A potential role of the nasopharyngeal (NP) microbiota has been hypothesized but to date, most of the studies have been focused on adults. We studied the NP microbiota modifications in children affected by SARS-CoV-2 infection showing a specific NP microbiome profile, mainly composed by pathobionts and almost missing protective anaerobic commensals. Moreover, in our study, specific microbial signatures appear since the first days of infection independently from SARS-CoV-2 viral load.

## INTRODUCTION

The human upper respiratory tract (URT) is colonized by a variety of different microbial communities (1). These communities are shaped by the specific anatomical location but also by the interaction with both intrinsic and external factors, such as aging, diseases, immune responses, olfactory function, and lifestyle habits (1). Furthermore, it is well known that the microbiota, colonizing the skin and the mucosal surfaces of the human body, can modulate the barrier function of its mucosa and the immune system with an active role in the host physiological and pathological processes (1).

Associations between changes in the nasopharyngeal (NP) microbiota and respiratory syncytial virus (RSV), influenza virus, or rhinovirus have been reported (2–5).

The SARS-CoV-2, a single-stranded positive-sense RNA virus belonging to the family of Coronaviridae (6), like other human coronaviruses, is mainly transmitted through the URT by aerosolized droplets carrying viral particles, that can bind to the angiotensin-converting enzyme 2 (ACE-2) receptor, whose expression is particularly high in the nasal and oral epithelial cells (6). Therefore, the relationship between SARS-CoV-2 infection and the NP microbiota has been thoroughly investigated. However, available data and results, limited and highly variable, are still mainly obtained from adult cohorts of COVID-19 patients (7–12).

Based on the evidence that the NP microbiota may promote the acquisition of several respiratory infections and have an impact on the evolution of their outcome (2–5) and that the adult and children have different disease courses of COVID-19, milder in children, we thought to fill the gap on the description of NP microbiota profile in children affected by COVID-19 and to determine whether any specific signature of their NP microbiota may actually be related to the disease onset.

## MATERIALS AND METHODS

### Study design and subjects' enrollment

Between 1 March 2020 and 30 September 2020, 78 children admitted to our hospital with signs and symptoms of suspected COVID-19 disease were consecutively enrolled in this study (13). Patients with NP swab positive for SARS-CoV-2, assayed by a molecular test, were considered as COVID-19 confirmed cases. Patients with a NP negative swab for SARS-CoV-2, assayed by molecular test, and with diagnosis different from COVID-19 (7 patients) were in any case included in the study and considered as a separate group (NO-COVID-19 cohort).

Patients affected by COVID-19 were stratified on the basis of disease severity according to the WHO clinical progression scale: (i) “mild,” without evidence of viral pneumonia or hypoxia; (ii) “moderate,” with clinical signs of non-severe pneumonia (cough or difficulty breathing, fast breathing, and/or chest in drawing) but without signs of severe pneumonia; (iii) “severe,” with clinical signs of pneumonia (cough or difficulty breathing) and with at least one of the other following signs: (a) central cyanosis or saturation of peripheral oxygen <90%; severe respiratory distress; general danger sign such as inability to breastfeed or drink, lethargy or unconsciousness, or convulsions; and (b) fast breathing (age <2 months: ≥60 breath/min; age 2–11 months: ≥50 breath/min; age 1–5 years: ≥40 breath/min) (14).

Age, gender, clinical, and routine laboratory characteristics were collected for each patient. One-hundred forty-five NP swabs from all patients were collected at three time points: *T*_0_, close to the admission, within 48–72 h; *T*_1_, 72 h to 7 days since the admission; and *T*_2_, at the discharge. All NP swab samples were stored at −80°C until processing. Both cohorts (COVID-19 and NO-COVID-19) were compared to each other and with healthy controls (controls, CTRLs). The CTRL cohort was composed by 59 healthy subjects, age-matched with patients, selected among patients having control visits for elective surgical procedures, and underwent a SARS-CoV-2 negative molecular test.

Local Ethical Committee approved the study, and written informed consent was obtained from parents and legal guardians of all participants (2083_OPBG_2020).

### Detection of SARS-CoV-2 on NP swabs

Nucleic acids were extracted using the STARMag Universal Cartridge kit (Seegene) in automated Nimbus IV platform and eluted in 100 µL of elution buffer. Real-time PCR was performed on CFX96 (Bio Rad Laboratories) with Allplex 2019-nCoV kit, using 5 µL of extracted RNA in a final volume of 25 µL. An internal control was included in each sample for checking the extraction efficiency and PCR inhibition. In every experimental session, a negative control was used to monitor carry-over contamination. The results were analyzed automatically using Seegene software (Seegene Viewer V2.0). Target genes were *envelope* (E), *RNA-dependent RNA polymerase* (RdRP), and *nucleocapsid* (N). Samples were considered positive when one or more genes were detected. Cycle threshold (*C*_T_) values were exploited to assess viral load (VL): *C*_T_ < 25 high VL; 25 < *C*_T_ < 30 medium VL; *C*_T_ > 30 low VL, according to UNI EN ISO 9001:2015 quality standard procedures.

### Bacterial DNA extraction from NP swabs and 16s rRNA targeted-metagenomics

DNA was extracted from NP swabs by EZ1 DNA Tissue Kit and biorobot EZ1 extractor following the manufacturer's instructions (Qiagen, Hilden, Germany). The 16S rRNA regions V3–V4 (~460 bp) were amplified following the MiSeq rRNA Amplicon Sequencing protocol (Illumina, San Diego, CA, USA). DNA libraries were indexed by using Nextera (Illumina) technology in a second amplification step. Finally, each library was cleaned, quantified with Quant-iT PicoGreen dsDNA Assay Kit (Thermo Fisher Scientific, Waltham, MA, USA), diluted to 4 nM concentrations, and pooled, before sequencing on Illumina MiSeq platform (Illumina) (13).

### Biocomputational and statistical analysis of NP microbiota profiles

For each sample, two fastq file were generated and analyzed using QIIME2. DADA2 plug in was used for reads' quality control, denoising, chimera removal, trimming, and construction of the amplicon sequence variant (ASV) table (15). The taxonomy was assigned by using a Naive Bayes model (q2-feature classifiers plug in) pre-trained on SILVA database (https://www.arb-silva.de/) (16). The Cum Sum Scaling (CSS) methodology was used for the ASV relative abundance normalization, after filtering out the unassigned reads (17). Skbio.diversity (Python 3.7 library) was used for the α- and β-diversity calculation, and for permutational analysis of variance (PERMANOVA test), respectively; the latter was applied on weighted and unweighted Unifrac, Bray–Curtis, and Euclidean distance matrices (18, 19) with 9,999 permutations to perform paired-comparison of sample groups. Principal coordinate analysis (PCoA) plots were used to illustrate the beta diversity of samples. The Shapiro–Wilk test was used to test normality. The constrained correspondence analysis (CCA) was used to measure the confounding factors by the R package “microbiomeMarker.”

To identify microbial markers associated with each group, linear discriminant analysis (LDA) effect size (LEfSe) analyses was used (20). An *α* value of 0.05 and an effect size threshold of 2 were used to identify the significant predicted microbial biomarkers. Unless otherwise stated, all ecological statistical analyses were performed using Python 3.7.

On Kruskal–Wallis test, three different levels of statistical significance were identified based on different *P* values (*P* ≤ 0.001) and false discovery rate (FDR) thresholds (*P* ≤ 0.05, *P* ≤ 0.001) (21).

Phylogenetic Investigation of Communities by Reconstruction of Unobserved States 2 (PICRUSt 2) (22), exploiting the Kyoto Encyclopedia of Genes and Genomes (KEGG) orthologs (KO) database were used to determine NP microbiome functional potential for COVID-19 patients and healthy subjects.

### Clustering analysis and machine learning

Heat maps based on hierarchical clustering of COVID-19 disease vs healthy status (CTRLs) groups represented with respect to ASVs content were performed by Pearson's correlation distance. On ASVs table at phylum (L2), family (L5), and genus (L6) were applied these filters: interquartile range (IQR) ≠ 0. Percentage of zeros ≥75% and fold-change (FC) ≠ 0; the variables that were totally unnecessary for classification were discarded. Then for each ASV the count of patients in each class with abundances >0, mean and SD of the abundances, and a Z-test with relative *P* value between the two classes were calculated.

Multiple machine learning (ML) models were trained for the classification tasks, by a 10-fold cross-validation with a train-test split of 70%–30%. To evaluate the model, the global and the single class accuracies were considered. The models tested were Logistic Regression, SGD Classifier, Logistic Regression CV, Hist Gradient Boosting Classifier, Random Forest Classifier, Extra Trees Classifier, Gradient Boosting Classifier, Bagging Classifier, Ada Boost Classifier, XGB Classifier, XGBRF Classifier, MLP Classifier, Linear SVC, SVC, Gaussian NB, Decision Tree Classifier, Quadratic Discriminant Analysis, K Neighbors Classifier, and Gaussian Process Classifier. An explain ability algorithm based on a permutation performance with 1,000 repetitions was followed.

## RESULTS

### Patient cohort, stratification, and sample collection

Seventy-one children with confirmed SARS-CoV-2 infection (i.e., COVID-19) and seven patients with negative SARS-CoV-2 test (i.e., NO-COVID-19) were included in this observational cohort study.

Demographic and clinical characteristics of COVID-19 and NO-COVID-19 cohorts are reported in Table 1.

**TABLE 1:** Demographic and clinical features

Variables		COVID-19	Non-COVID-19
Age			
Median (IQR^*a*^)		6.5 years (IQR 1.2–11)	3.5 years (IQR 1.4–5.3)
Min		8 days	1 year
Max		17.7 years	6 years
Sex			
Male		38 (53%)	2 (29%)
Comorbidities		5 (7%)	1 (15%)
Symptoms at the admission			
Fever		37 (52%)	6 (86%)
Respiratory symptoms			
Cough		16 (23%)	4 (57%)
Shortness of breath		4 (6%)	1 (15%)
Rhinorrhea		3 (4%)	0
Gastrointestinal symptoms			
Diarrhea		9 (13%)	2 (29%)
Vomit		4 (6%)	1 (15%)
Disease severity			
Asymptomatic		13 (18%)	NA
Mild		53 (75%)	NA
Moderate		5 (7%)	NA
Blood result			
Lymphopenia		18 (25%)	0
CRP^*b*^, mg/dL (IQR)		0.07 (IQR 0.05–0.33)	7.74 (IQR 4.13–11)
VL^*c*^	High	22	NA^*d*^
	Medium	13	
	Low	15	
Radiological results			
Suggestive of viral pneumonia		10 (14%)	NA
Co-infection			
Viral co-infection		1 (1.5%)	NA
Bacterial co-infection		3 (4%)	NA

^*a*^ IQR, interquartile range.

^*b*^ CRP, C reactive protein.

^*c*^ VL, viral load.

^*d*^ NA, not available.

There were 38 male and 33 female patients with COVID-19 with a median age of 6.5 years. Among them, four were affected by a coinfection: one had human herpes virus 6 (HHV6), two had a urinary tract infection (UTI), and one had gastroenteritis by *Campylobacter jejuni*. Moreover, five children had comorbidities such as a genetic syndrome, autism, Kikuchi–Fujimoto syndrome, connective tissue disorder, and psychomotor retardation. Only 18 (25.3%) patients received antibiotic therapy during the admission.

Based on VL, our population was stratified as follows: 22 patients were characterized by high VL, 13 by medium VL, and 15 by low VL, while for 21 patients *C*_T_ values were not available.

Overall, 131 NP swabs from patients with COVID-19 were collected, including 71 at the admission (*T*_0_), 42 at 7 days since the admission (*T*_1_), and 18 at the discharge (*T*_2_).

The NO-COVID-19 cohort included seven patients with a median age of 3.5 years. Six patients had lower respiratory infections, while only one patient reported URT infections (Table 1).

### Dysbiosis of NP microbiota in COVID-19 children

**Fig 1:**
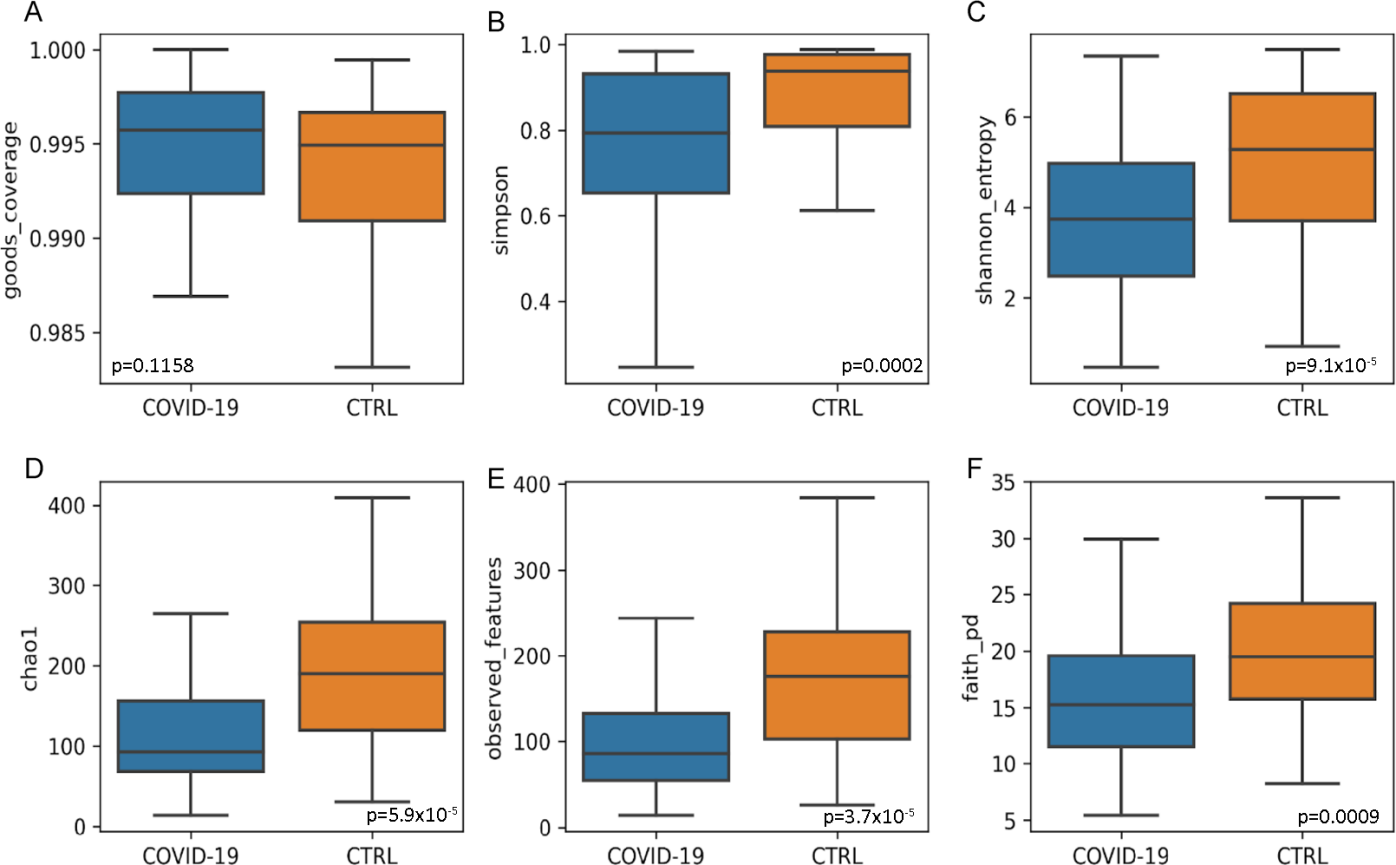
NP microbiota ecology. Alpha-diversity representation of COVID-19 and CTRLs groups based on Goods coverage (**A**), Simpson (**B**), Shannon (**C**), Chao-1 (**D**), observed features (**E**), Faith PD (**F**) indices. *P* values were obtained by Kruskal–Wallis test.

Prior to analyze the NP microbiota of our patient cohorts, we evaluated the presence of comorbidities, infections with other pathogens, disease severity, antibiotic and antiviral treatments, and steroid assumption as possible confounders in our analyses, by the CCA. None of these variables influenced the NP composition (Table S1). To assess the overall differences of microbial community structures, we evaluated the ecological richness for NP microbiota of COVID-19 and CTRL cohorts by α-diversity (Fig. 1). A significant α-diversity reduction was observed for the COVID-19 cohort compared with CTRLs (*P* value < 0.05) (Fig. 1).

**Fig 2:**
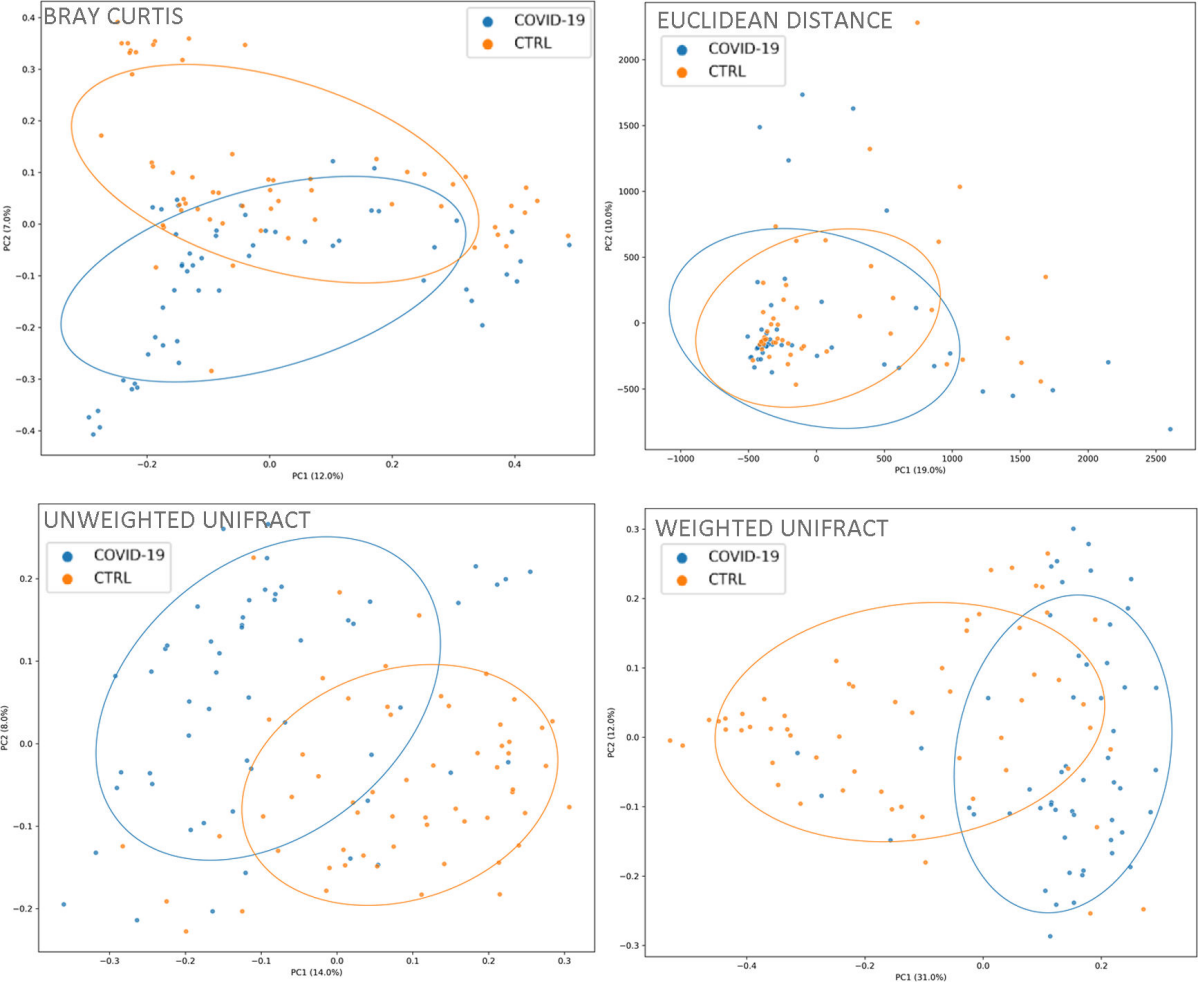
Beta-diversity representation of COVID-19 and CTRLs groups, based on Bray–Curtis, Euclidian distance, and unweighted and weighted UniFrac algorithms. *P* values were obtained by PERMANOVA (*P* value < 0.05 for all analyses).

Beta-diversity, performed by Bray–Curtis, Euclidean distance, and unweighted and weighted UniFrac algorithms, showed for NP microbiota of COVID-19 and CTRLs two separate clusters (*P* value < 0.05) (Fig. 2).

LDA performed on COVID-19 and CTRLs underlined several statistically significant differences in bacterial taxa abundance of NP microbiota. Differential distributions, observed at phylum and family levels are reported in Fig. S1, panels A and B. In particular, in the NP microbiota of the COVID-19 cohort compared with CTRLs, Proteobacteria, Fusobacteriota, Deinococcota, Thermotogota, and Actinobacteria were statistically increased, whereas Verrucomicrobiota, Firmicutes, Bacteroidota, and Euryarchaeota were statistically reduced in the NP microbiota of COVID-19 patients (Fig. S1, panel A). These results were confirmed by the analysis at family rank, in which clear was the increase of Staphylococcaceae, Gemellaceae, Streptococcaceae, Lactobacillaceae, Enterococcaceae, Bacillaceae, Flavobacteriaceae, Prevotellaceae, Micrococcaceae, Burkholderiaceae Pasteurellaceae, Enterobacteriaceae, Neisseriaceae, Veillonellaceae, Enterococcaceae, Moraxellaceae, and Micrococcaceae in the NP microbiota of COVID-19 cohort, while a statistically significant reduction was reported for Ruminococcaceae, Lanchnospiraceae, Akkermansiaceae, Oscillospiraceae, Bacteroidaceae, and Bifidobacteriaceae, compared to CTRLs (*P* value FDR < 0.001) (Fig. S1, panel B).

**Fig 3:**
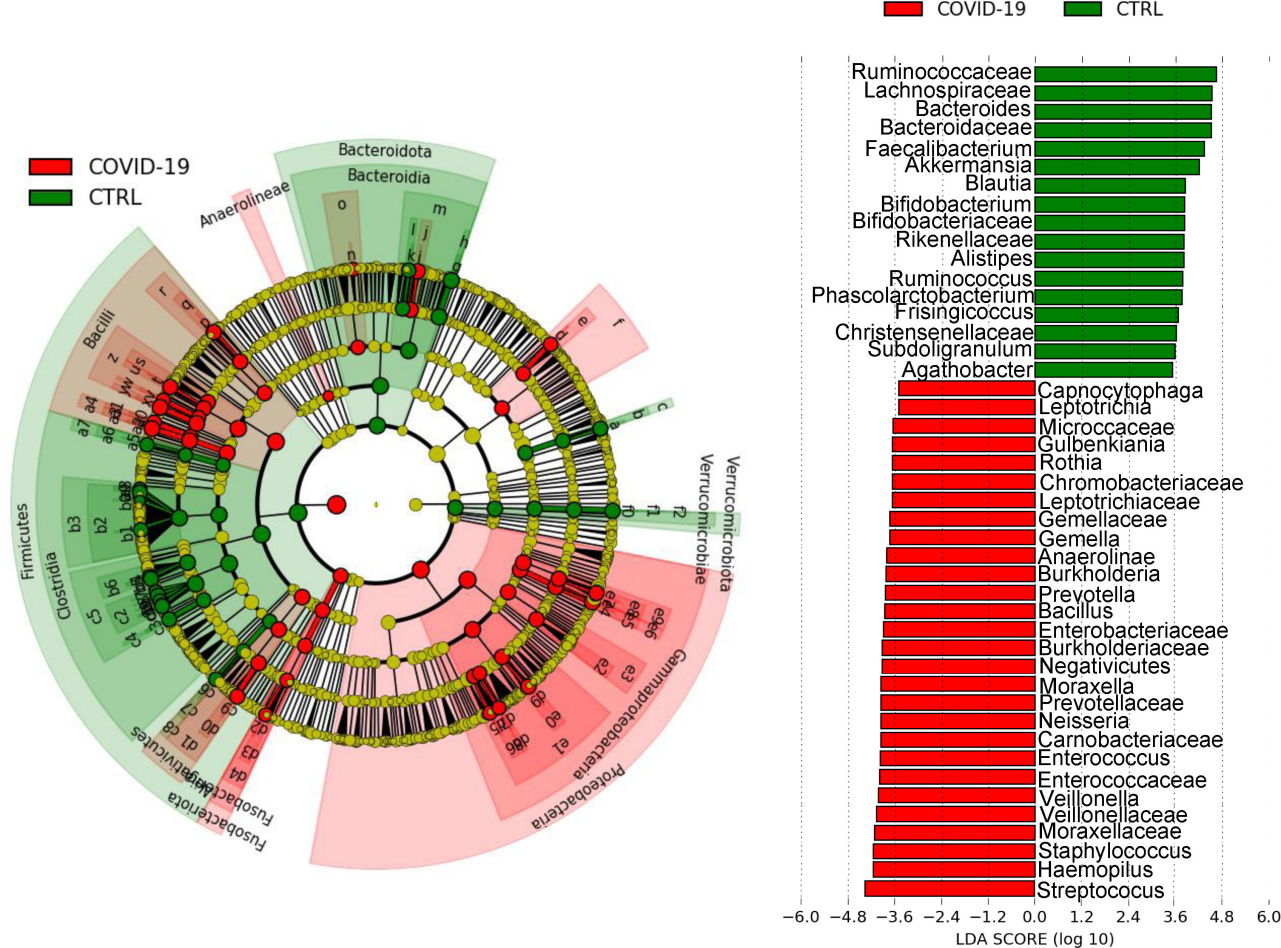
LEfSe cladogram and plot representation of taxa differences obtained for the NP microbiota of COVID-19 and CTRLs groups. Bar and nodes highlighted in red and green were significantly more abundant more abundant in COVID-19 and CTRLs, respectively (*P* value FDR < 0.001).

At genus rank level, the NP microbiota of COVID-19 cohort was enriched in Streptococcus in *Streptococcus*, *Haemophilus*, *Staphylococcus*, *Veillonella*, *Enterococcus*, *Neisseria*, *Moraxella*. Enterobacteriaceae, *Gemella*, *Bacillus*, and deprived in *Faecalibacterium*, *Akkermansia*, *Blautia*, *Bifidobacterium*, *Ruminococcus*, and *Bacteroides*, compared to CTRLs (*P* value FDR < 0.001) (Fig. 3).

### Correlation between bacterial taxa revealed a specific NP microbiota associated to COVID-19 disease

Pearson's correlations among ASVs revealed a particular NP microbiota of COVID-19 patients. At phylum level, Actinobacteria, Proteobacteria, Fusobacteriota, and Campilobacterota were positively correlated with patients affected by SARS-CoV-2 infection, whereas Firmicutes, Verrucomicrobiota, and Bacteroidota were negatively correlated with the infection (Fig. S2, panel A).

At family level, the hierarchical cluster showed that Micrococcaceae, Neisseriaceae, Pausteurellaceae, Streptococcaceae, Enterococcaceae, Staphylococcaceae, and Corynebactericeae positively correlated with COVID-19, whereas Lachnospiraceae, Oscillospiraceae, Ruminococcaceae, and Bacteroidaceae were negatively correlated (Fig. S2, panel B).

**Fig 4:**
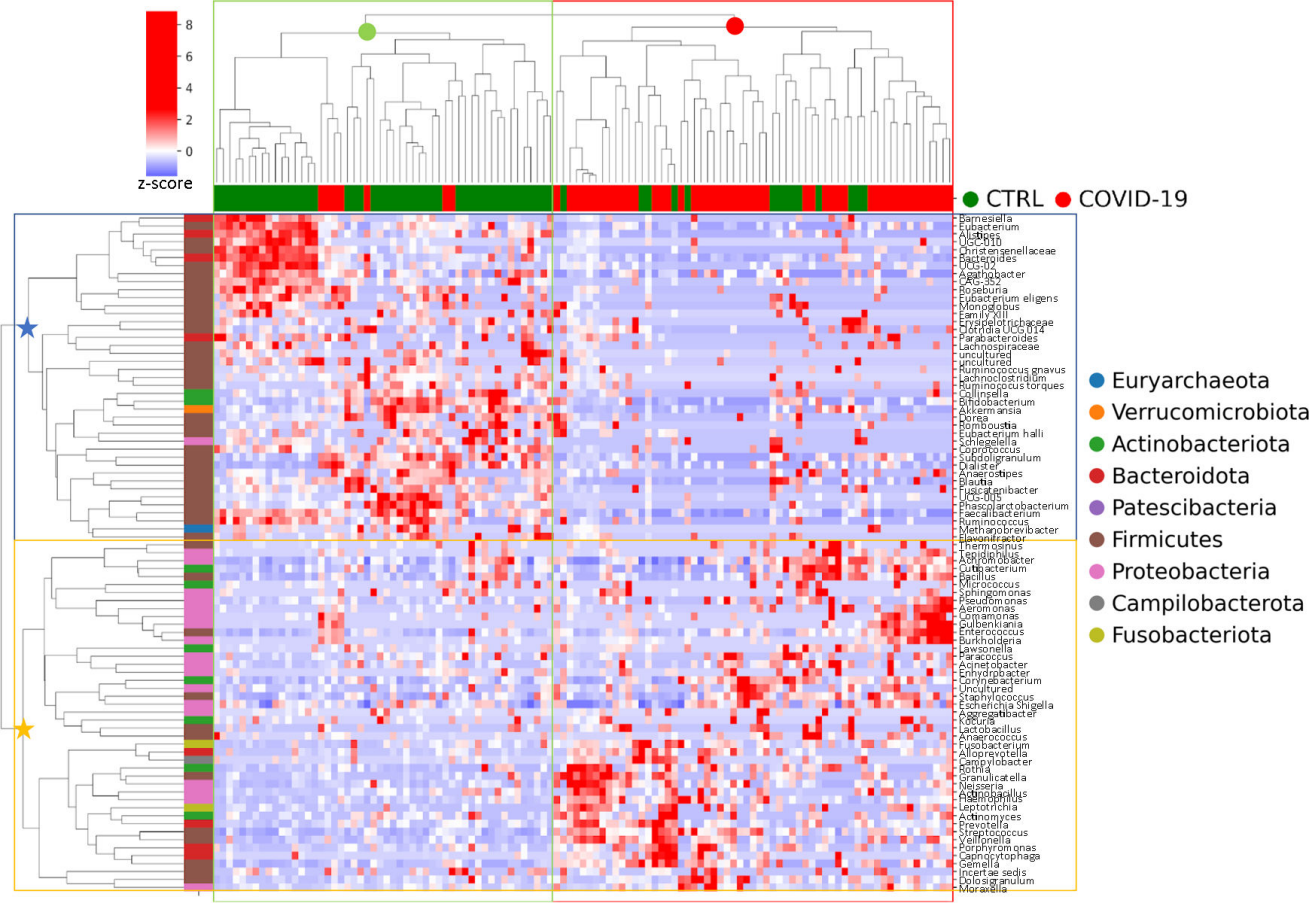
Graphical representation of hierarchical analysis of bacterial distribution at genus level of COVID-19 and CTRLs groups. In the heatmap, the hierarchical complete linkage dendrogram is based on the ASVs Pearson’s correlation coefficient. The color scale characterizes the *Z*-score for each variable: red, high level; blue, low level. Red circle, cluster mainly composed by COVID-19; green circle, cluster mainly composed by CTRL; blue star, cluster composed by bacterial taxa negatively correlated with the disease; yellow star, cluster composed by bacterial taxa negatively correlated with SARS-CoV-2 infection.

At genus level, a clear-cut separation of NP microbiota communities between COVID-19 and CTRL groups was reported. In particular, among others, *Pseudomonas*, *Comamonas*, *Guilberkiana*, *Burkholderia*, *Rothia*, *Granulicatella*, *Neisseria*, *Actinobacillus*, *Haemophilus*, *Prevotella*, *Streptococcus*, *Veillonella*, *Porphyromonas Gemella*, and *Moraxella* were positively correlated with SARS-CoV-2 infection. Conversely, *Barnesiella*, *Alistipes*, *Bacteroides, Roseburia*, *Ruminococcus torques, Ruminococcus gnavus*, *Collinsella*, *Bifidobacterium*, *Akkermansia*, *Dorea*, *Dialister*, *Anaerostipes*, *Blautia*, *Faecalibacterium*, and *Methanobrevibacter* were negatively correlated with the infection (Fig. 4).

### COVID-19-associated NP microbial markers

**Fig 5:**
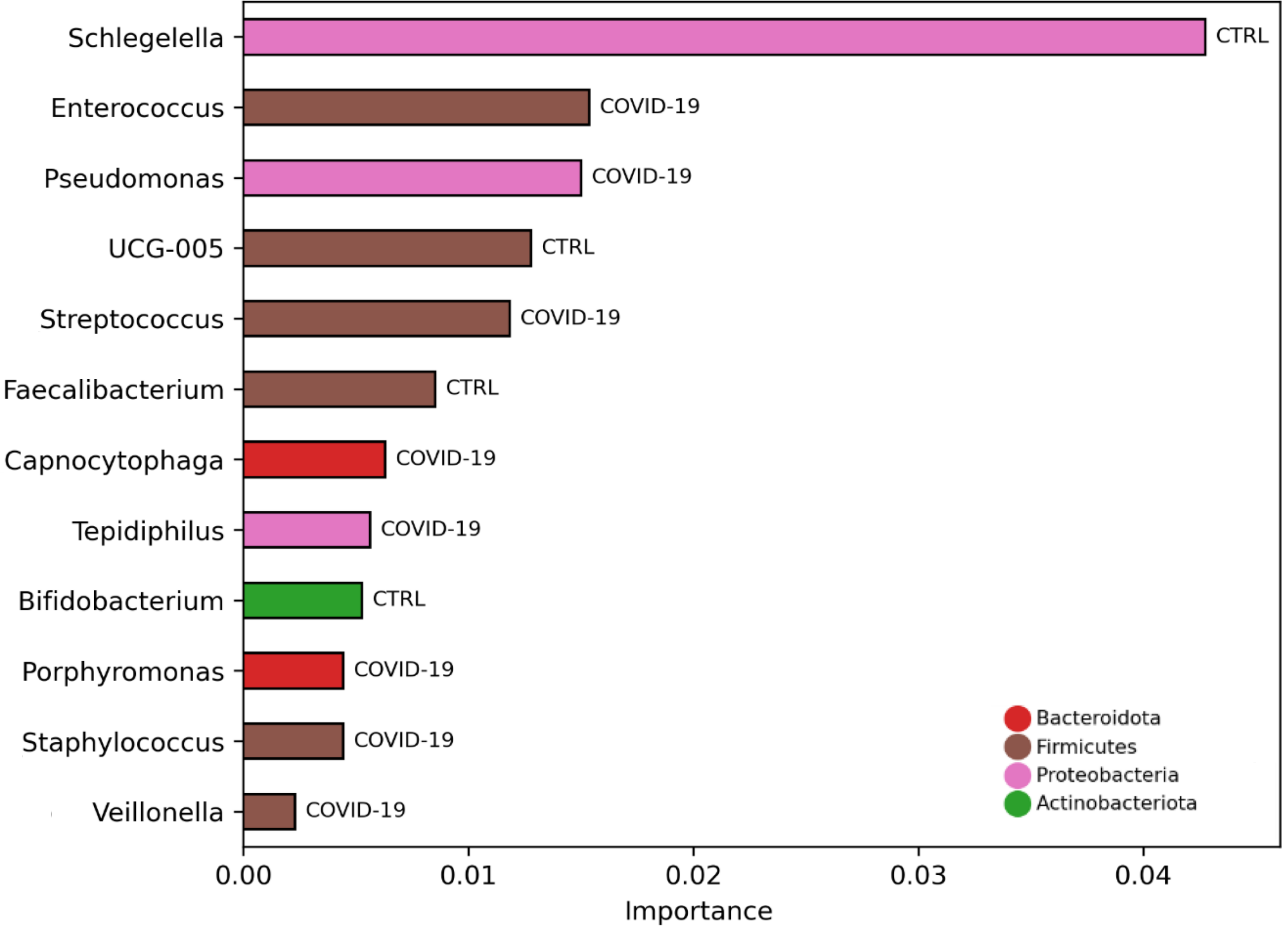
Important bacterial taxa selected by model classification analysis. The bars represent the importance scores of each ASV in the prediction of models.

To investigate if NP microbiota profile was predictive of COVID-19 disease, ML models were exploited (Table S2). By this approach *Enterococcus*, *Pseudomonas*, *Streptococcus*, *Capnocytophaga*, *Tepidiphilus*, *Porphyromonas*, *Staphylococcus*, and *Veillonella* were identified as COVID-19 disease-associated NP microbial markers (Fig. 5; Table S3).

The NP microbiota had the capability to classify 100% of both COVID-19 and CTRLs NP microbiota by the models Hist Gradient Boosting, Extra Trees, and MLP Classifiers (Table S2).

### Functional profiling of COVID-19-associated NP microbiota

**Fig 6:**
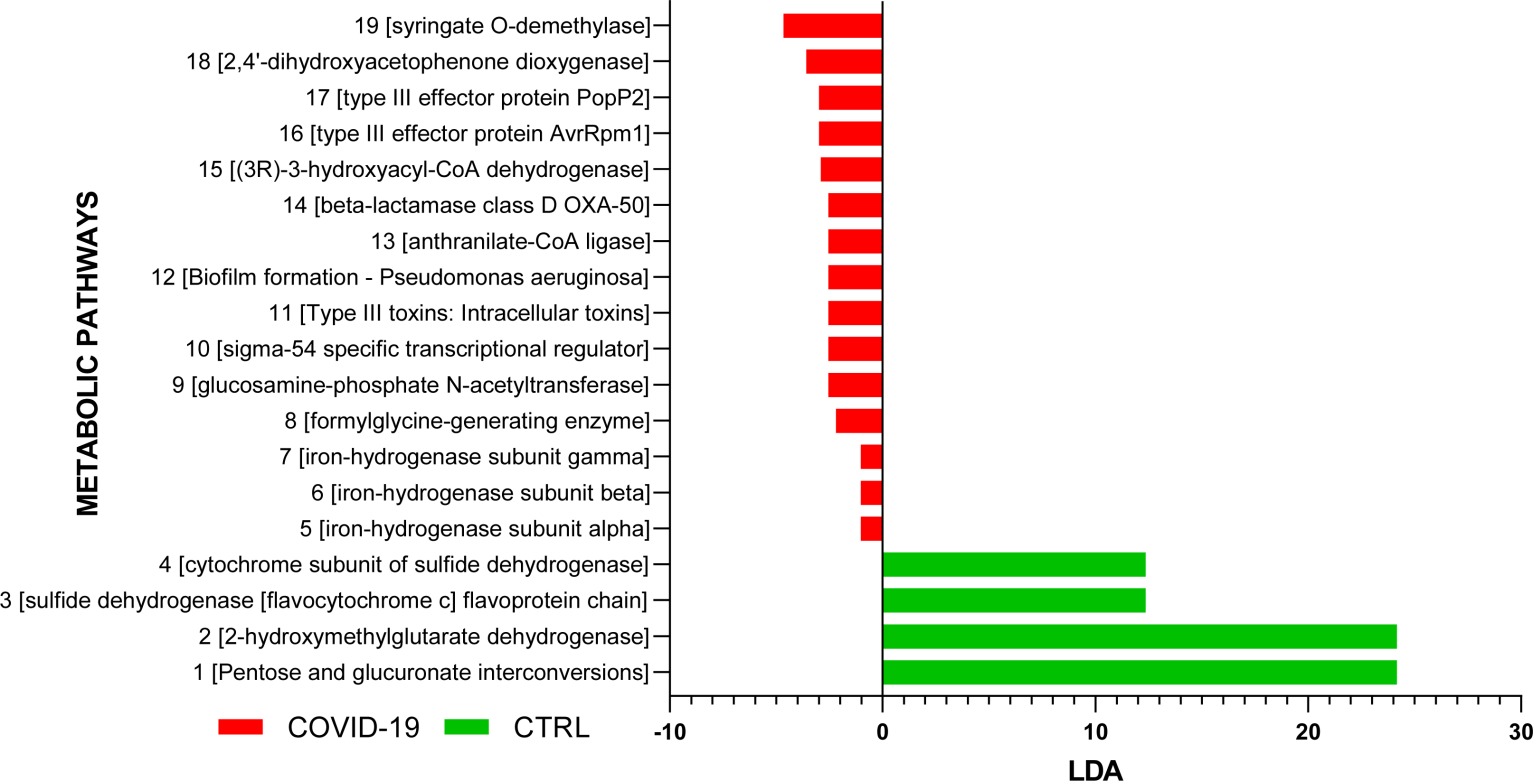
Functional analysis of the NP microbiota of COVID-19 patients and CTRLs. PICRUSt was used to infer the functional content of the NP microbiota based on 16S rRNA metataxonomic data. Log2 fold change representation of the abundances of functional pathways shows significant difference between COVID-19 and CTRLs groups. Red and green colors indicate the increase and the decrease of abundances of pathways in COVID-19 affected patients NP.

Functional profile of the NP microbiota, inferred by PICRUSt two computation, was characterized by 3.654 differentially expressed metabolic pathways in the pairwise comparison between COVID-19 patients and CTRLs; 15 and 4 were unique to each COVID-19 and CTRLs NP microbiota, respectively (Fig. 6).

### The impact of SARS-CoV-2 infection, its persistence, and VL on the COVID-19 NP microbiota ecology

Last, we evaluated the impact of SARS-CoV-2 presence, persistence, and VL on NP microbiota modulation. Comparing the ecology of NP microbiota of COVID-19 and NO COVID-19 patients (Fig. S3, panels A and B), we did not find any statistically significant difference between these two cohorts. However, the pairwise comparison revealed the increase of *Dialister* and the decrease of *Pseudomonas*, *Fusobacterium*, *Paracoccus*, *Sphingomonas*, and *Alloprevotella* in COVID-19 patients (Fig. S4). During patients' hospital stay, through the *T*_0_*–T*_2_ time-course points, the NP microbiota ecology of the COVID-19 cohort was not affected by any modulation (Fig. S5, panels A and B).

Moreover, COVID-19 NP microbiota comparisons, based on high, medium, and low VL value clustering, associated to *C*_T_ values of SARS-CoV-2 real-time assay, did not provide any statistically significant difference (Fig. S6, panels A and B).

## DISCUSSION

To date, only few studies have described the NP microbiota of children affected by SARS-CoV-2 infection and the present is the second largest cohort of COVID-19 pediatric patients (23) studied so far. Our results suggest that NP microbiota of COVID-19 pediatric patients changes since the first days of infection and has statistically significant differences in terms of richness (i.e., α- and β-diversity) and composition when compared with healthy subjects. Despite the variability of data obtained for NP microbiota of adult cohorts, our results agreed with the study conducted by Zhang et al. describing a reduced α-diversity in the COVID-19 patients (11).

However, similar Shannon and Simpson indexes of NP microbiota from 22 children with SARS-CoV-2 RNA positivity, compared with children with no RNA detection, was reported by the study of Rocafort et al. (24). Moreover, Hurst et al. described the absence of correlation between α-diversity and the presence of COVID-19 symptoms in SARS-CoV-2 infected children (23). Candel et al. discussed that the majority of studies highlighted statistically significant changes in NP microbiota richness when comparing COVID-19 patients to healthy CTRLs (25), and indeed studies that did not observe any difference concerning NP microbiota diversity were all conducted for very small cohorts of patients, also heterogenous in term of timing of SARS-CoV-2 infection (25).

In terms of NP microbiota global distribution then, Tchoupou Saha et al. reported *Corynebacterium propinquum/pseudodiphtericum, Moraxella catarrhalis*, *Bacillus massiliamazoniensis*, *Anaerobacillus Alkalidiazotrophicus*, *Staphylococcus capitis* subsp. *capitis*, and *Afipia birgiae* as specific COVID-19 microbial signature of the URT for adult subjects (5). Rosas-Salazar et al. described *Peptoniphilus lacrimalis*, *Campylobacter hominis*, *Prevotella copri*, and *Anaerococcus* as more abundant in the URT of SARS-CoV-2-infected adult patients and especially in those with high VL (10). Conversely, De Maio et al. showed no major differences in the NP microbiota composition of COVID-19 adult patients compared to uninfected CTRLs (7). In another study, Bacteroidetes and Firmicutes were significantly reduced, whereas Proteobacteria was enriched in the URT of COVID-19 adult patients (26).

Instead, in a pediatric cohort *Moraxella* was described as a specific signature of the nasal microbiota of CTRLs, suggesting its protective role against COVID-19 in childhood (27).

In our cohort, *Streptococcus*, *Haemophilus*, *Staphylococcus*, *Veillonella*, *Enterococcus*, *Neisseria*, *Moraxella*, Enterobacteriaceae, *Gemella*, and *Bacillus* were statistically significantly more abundant in COVID-19 patients, while *Faecalibacterium*, *Akkermansia*, *Blautia*, *Bifidobacterium*, *Ruminococcus*, and *Bacteroides* were less reported.

The reduction of anaerobic bacteria and the increase of pathobionts in the NP microbiota have already been reported by other studies, describing other viral respiratory tract infections (10, 28, 29). Gauthier et al. highlighted, in an adult SARS-CoV-2-infected group, that the NP microbiota was dominated by common nasal pathobionts and opportunistic pathogens such as *Haemophilus influenzae*, *Staphylococcus haemolyticus*, and *Staphylococcus aureus* (30). Moreover, according to our results, the author did not find significant differences for α- and β-diversity of the NP microbiota composition stratifying samples on the basis of *C*_T_ value; these data seem to suggest that the impact of SARS-CoV-2 on NP microbiota is independent from VL. The evidence of a reduction in commensal bacterial species and an enrichment of opportunistic Gram-negative pathogens, frequently associated with multidrug resistance, was observed also in bronchoalveolar lavage of critically ill adult patients affected by COVID-19 (31).

Moreover, it has been shown, during viral infections, that the increase of *Haemophilus* and *Streptococcus* leads to the upregulation of the adhesion receptors for viral entry (32). Indeed, the enrichment of various opportunistic pathogens during NP microbiota dysbiosis processes or impaired host immunity might promote the entry of virus leading to secondary infection or increase of disease symptoms.

Interestingly, different studies have confirmed a differential NP microbiota profile in SARS-CoV-2 infected children with respiratory symptoms compared to NP microbiota of subjects without symptoms (23, 29). Moreover, it has been reported that a strong association exists between age and NP tract composition, the latter potentially affecting the identification of specific COVID-19 bacterial biomarkers or disease symptoms (23, 29).

In our study, the median age of patient and CTRLs cohorts was matching (6.5 years old), and therefore the age as a confounding factor of NP microbiota profiling was excluded, hence reinforcing the detected bacterial biomarkers associated to SARS-CoV-2 infection.

Moreover, beneficial microbes, such as *Bifidobacterium* and other butyrate-producing bacteria, as *Faecalibacterium*, characterized by anti-inflammatory effects, were depleted in the URT microbiota of our COVID-19 pediatric patients (1).

In the end, we evaluated the impact of SARS-CoV-2, virus persistence, and VL on the NP microbiota ecology, showing an absent influence of these variables on NP microbiota composition.

Finally, 15 predicted functional pathways were expressed only in by the NP microbiota of COVID-19 patients. Interestingly, as unique KEGG pathways, a “beta-lactamase class D OXA-50” involved in beta-lactam resistance and a pathway related to “Biofilm formation for *Pseudomonas aeruginosa*” were computed. The same results were obtained by Haiminen et al. who described the “carbapenem biosynthesis” pathway increased in nasal swabs of adult COVID-19 patients (33). Also, Xu et al. found the “Biofilm formation-*P. aeruginosa*” pathway significantly enriched in NP swabs of COVID-19 pediatric patients (34).

We can summarize our findings in three main points: (i) as in adults, the NP microbiota composition of children affected by SARS-CoV-2 has a unique profile compared to healthy controls, (ii) the changes in the NP microbiota when SARS-CoV-2 is detected are independent from the viral load and persist during the course of the disease, and (iii) NP microbiota is COVID-19 children is functionally linked to pathway involved in antimicrobial resistant, as in adult population.

Despite the large cohort of COVID-19 pediatric patients enrolled in this study, the small sample size of the NO-COVID cohort, probably due to absence of other respiratory virus circulation, hampered *de facto* their comparison, impeding to perform a fulfilling NP microbiota characterization, depending by presence or absence of SARS-COV-2.

Moreover, our COVID-19 cohort was composed mainly on the patients with mild disease and therefore the comparison based on disease severity was not feasible.

### Conclusion

All together, these data support the hypothesis that pediatric COVID-19 patients have a specific NP microbiome profile, principally composed by pathobionts and missing of protective anaerobic commensals, acting as beneficial short chain fatty acids (SCFA) producing bacteria. Moreover, COVID-19-related microbial signatures appear since the first days of infection and are independent from the SARS-CoV-2 VL. Our data suggest that the NP microbiota may have a specific disease-related signature since infection onset without changes during disease progression, and regardless of the SARS-CoV-2 VL.

## Data Availability

All raw sequencing reads are available in the NCBI BioProject database (PRJNA993438).

## SUPPLEMENTAL MATERIAL

Supplemental material spectrum.03009-23-s0001.docx Figures S1 to S6; Tables S1 to S3.

ASM does not own the copyrights to Supplemental Material that may be linked to, or accessed through, an article. The authors have granted ASM a non-exclusive, world-wide license to publish the Supplemental Material files. Please contact the corresponding author directly for reuse.
