# Supplementary material for: Exploring nasopharyngeal microbiota profile in children affected by SARS-CoV-2 infection
Source: Microbiol Spectr. 2024 Jan 30;12(3):e03009-23. doi: 10.1128/spectrum.03009-23 (PMC10913489; doi:10.1128/spectrum.03009-23)
Supplement: Supplemental material — Figures S1 to S6; Tables S1 to S3. [file spectrum.03009-23-s0001.docx]

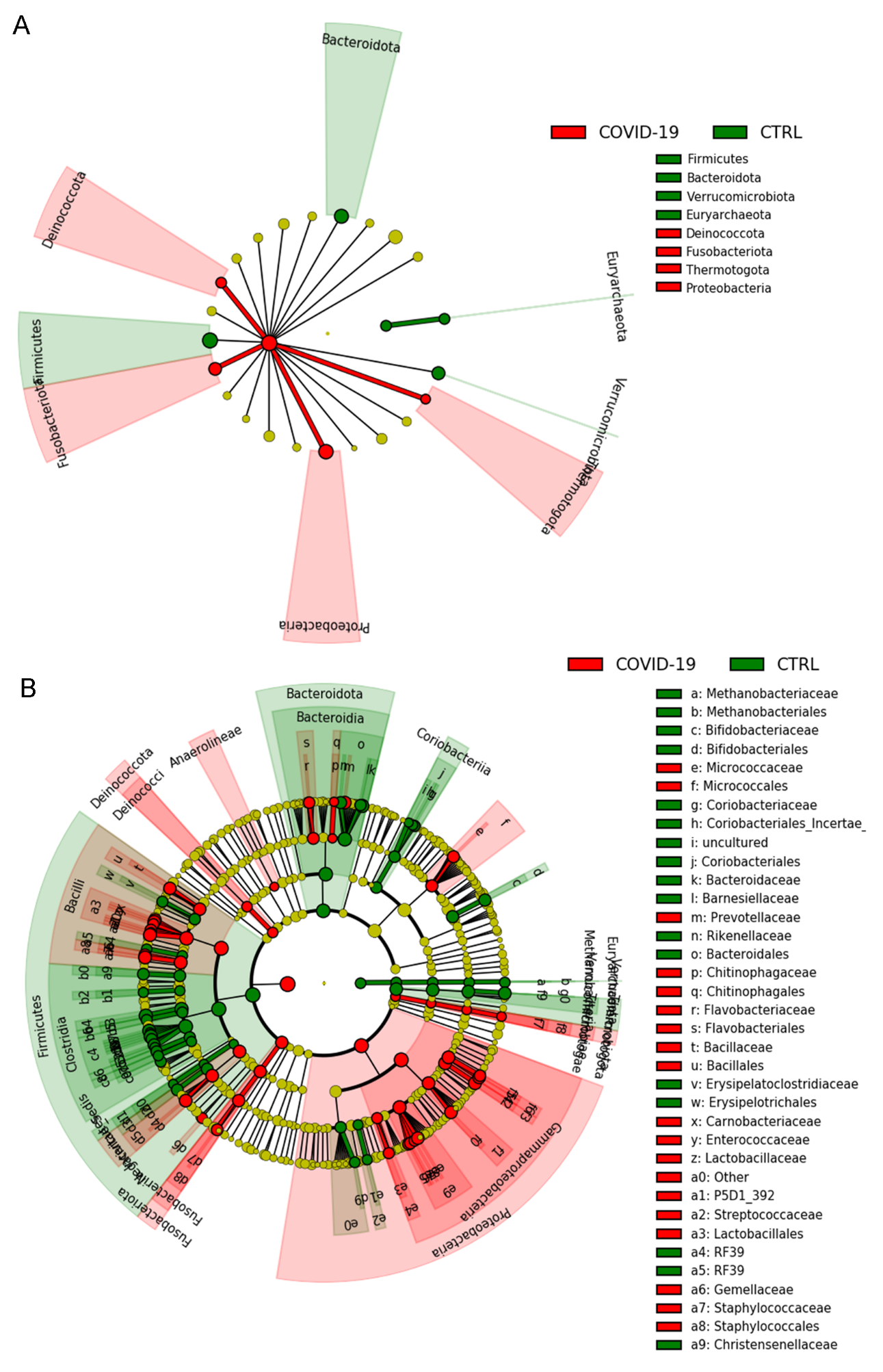


Figure S1. Linear discriminant analysis (LDA) Effect size (LEfSe) identified taxa that differentially characterize COVID-19 and CTRL groups at phylum (panel A) and family (panel B) levels.

**
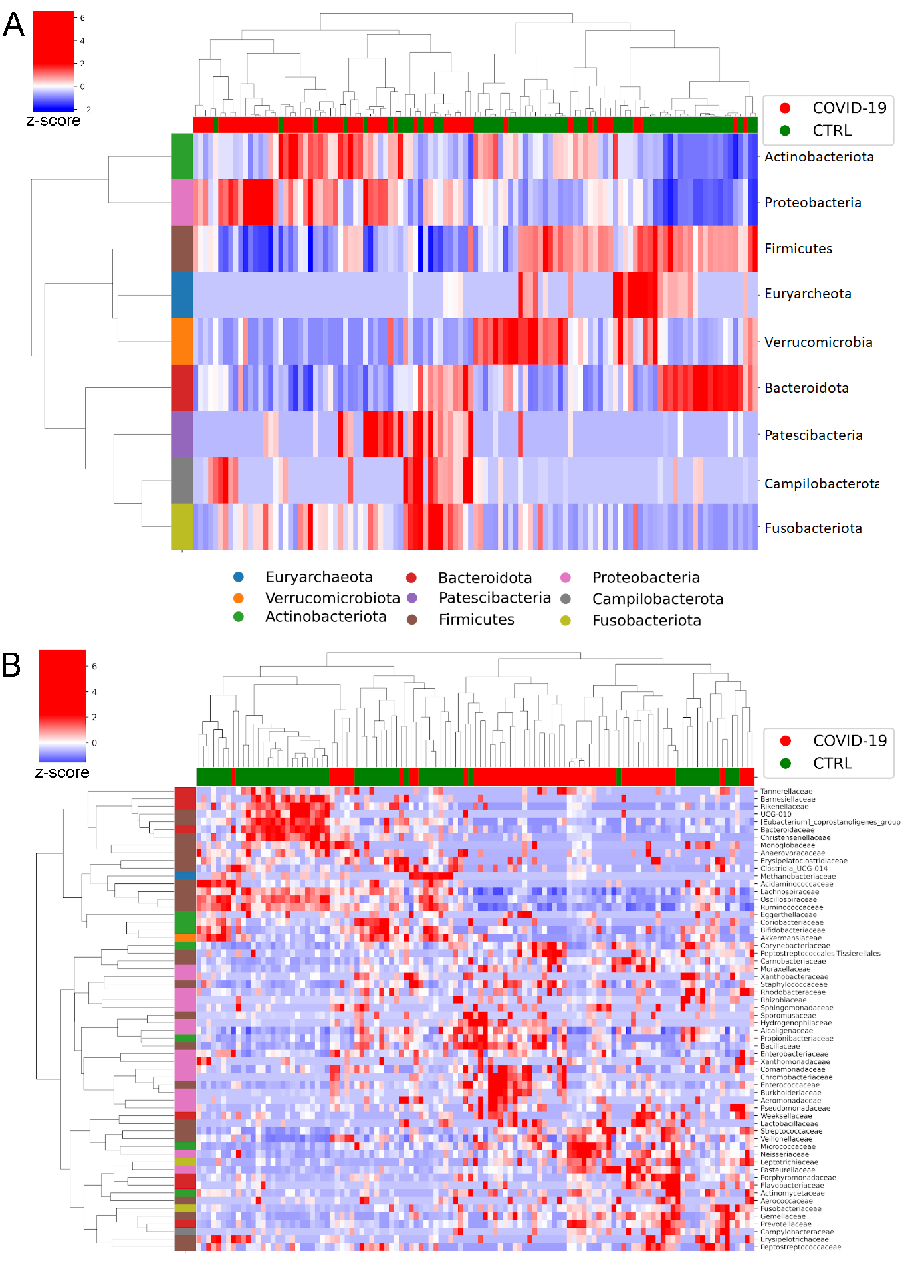
**

**Figure S2.** Graphical representation of hierarchical analysis of phylum (A) and family (B) distribution for the NP microbiota of COVID-19 and CTRLs groups, filtered by a t-test between classes with *p*-value <0.05. In the heatmap, the hierarchical complete linkage dendrogram is based on the phylum Pearson’s correlation coefficient. The colour scale characterizes the Z- score for each variable: red, high level; blue, low level.


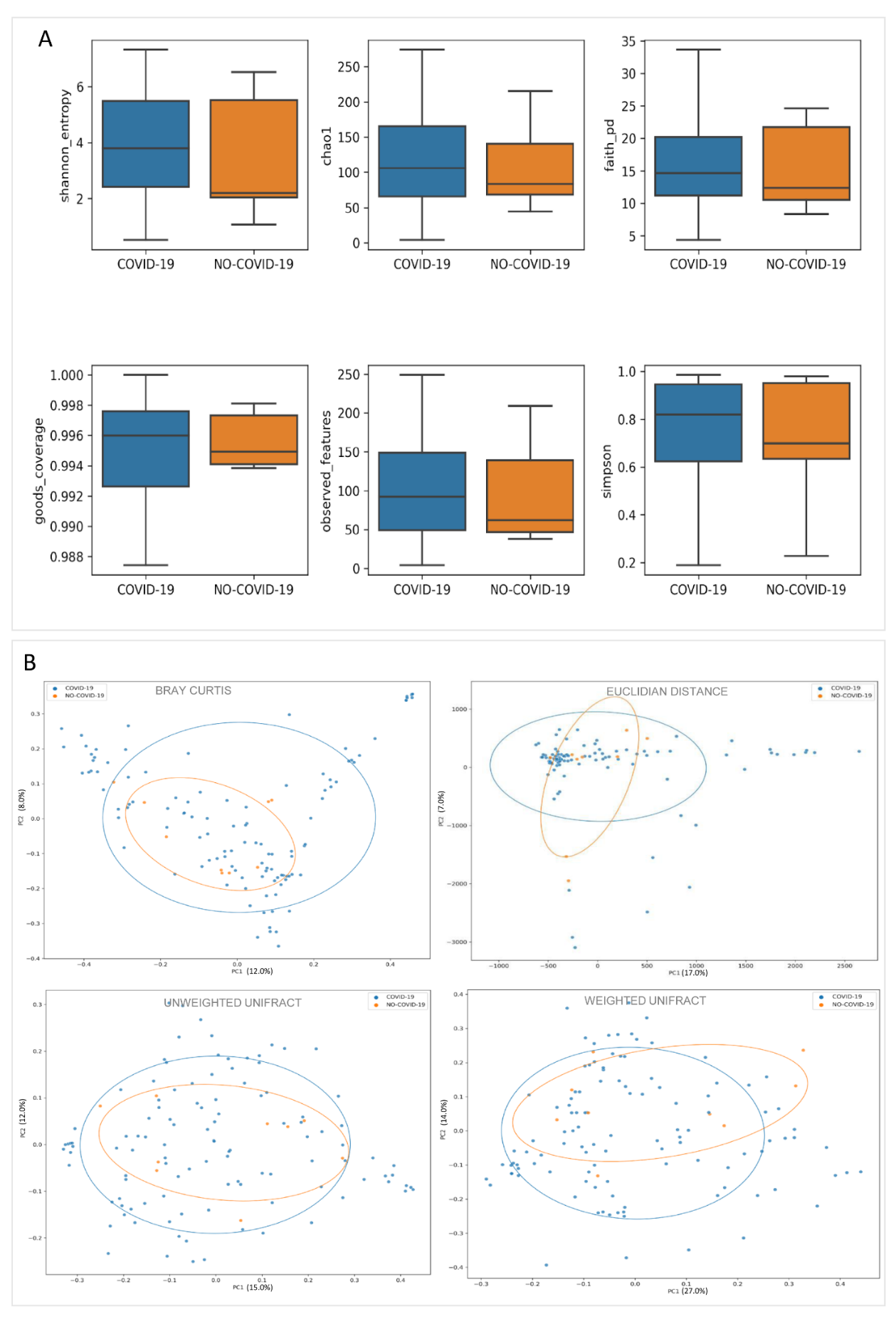


**Figure S3.** Evaluation of the α-diversity among COVID-19 and NO-COVID-19 based on Shannon, Chao-1, Faith PD, goods coverage, observed features, and Simpson indexes (**A**). Beta-diversity analyses, performed by Bray-Curtis, Euclidian distance, unweighted and weighted UniFrac algorithms for COVID-19 and NO-COVID-19 (**B**).


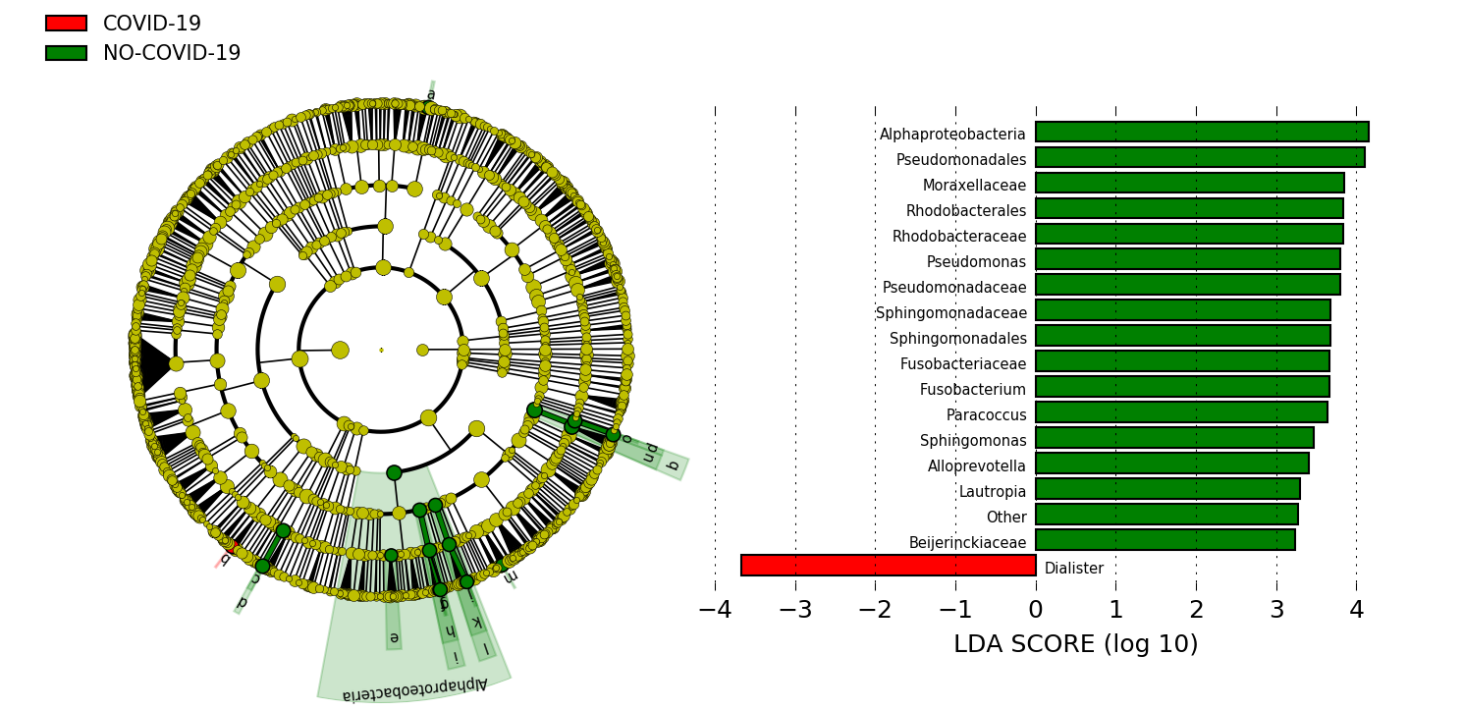


**Figure S4.** **LEfSe cladogram and plot reporting taxonomic differences between COVID-19 and NO-COVID-19.** Taxa and nodes highlighted in red and green were significantly more abundant in COVID-19 and NO-COVID-19, respectively.


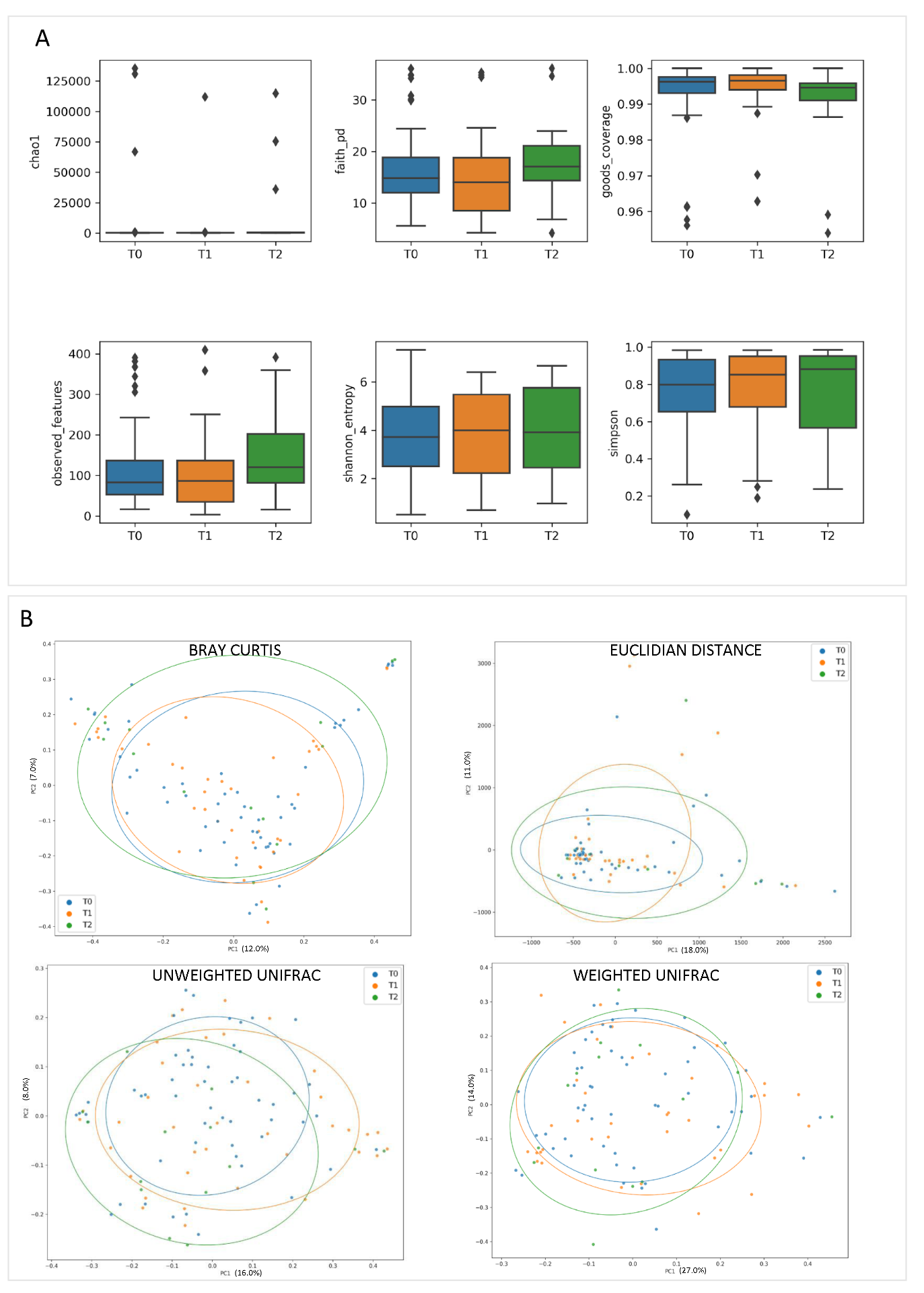


**Figure S5.** Evaluation of the α-diversity among COVID-19 at T_0_, T_1_ and T_2_ based on Chao-1, Faith PD, goods coverage, observed features, Shannon and Simpson indexes (**A**). Beta-diversity analyses, performed by Bray-Curtis, Euclidian distance, unweighted and weighted UniFrac algorithms for COVID-19 at T_0_, T_1_ and T_2_ (**B**).

**
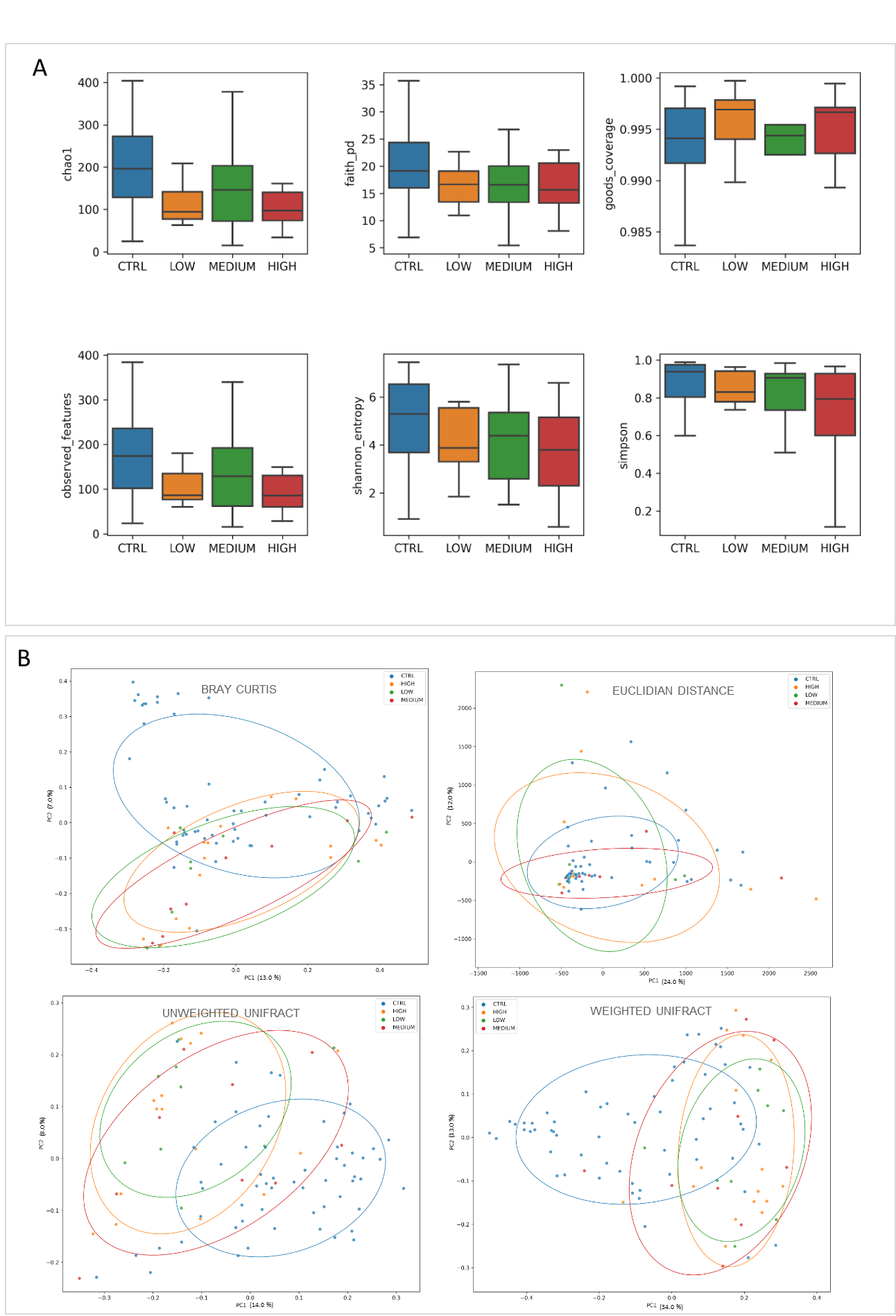
**

**Figure S6.** Evaluation of the α-diversity among COVID-19 grouped on SARS-CoV-2 viral load and CTRLs. Chao-1, Faith PD, goods coverage, observed features, Shannon, Simpson indexes are reported in panel A. Beta-diversity analyses, performed by Bray-Curtis, Euclidian distance, unweighted and weighted UniFrac algorithms for COVID-19 grouped on SARS-CoV-2 viral load CTRLs.

**Table S1. Analysis of confounding factors.** The constrained correspondence analysis (CCA) was used to identify possible confounding factors of microbiota profiling.

| Confounder | pseudo_F | pvalue |
| --- | --- | --- |
| Comorbidities | 1.4261 | 0.184 |
| Infections with other pathogens | 1.8755 | 0.054 |
| Disease severity | 1.4125 | 0.115 |
| Antibiotic treatments | 1.3022 | 0.165 |
| Antiviral treatments | 0.4526 | 0.808 |
| Steroid assumption | 0.8305 | 0.496 |

**Table S2.** Classification models applied to discriminate COVID-19 and CTRLs subjects based on NP microbiota composition.

| **Model** | **Score** | **Score COVID-19** | **Score CTRL** |
| --- | --- | --- | --- |
| Dummy Classifier | 0.50 | 0.00 | 1.00 |
| Logistic Regression | 0.95 | 0.91 | 1.00 |
| SGD Classifier | 0.95 | 0.91 | 1.00 |
| Logistic Regression CV | 0.91 | 0.82 | 1.00 |
| **Hist Gradient Boosting Classifier** | **1.00** | **1.00** | **1.00** |
| Random Forest Classifier | 0.92 | 1.00 | 0.83 |
| **Extra Trees Classifier** | **1.00** | **1.00** | **1.00** |
| Gradient Boosting Classifier | 0.74 | 0.82 | 0.67 |
| Bagging Classifier | 0.83 | 1.00 | 0.67 |
| Ada Boost Classifier | 0.96 | 1.00 | 0.92 |
| **MLP Classifier** | **1.00** | **1.00** | **1.00** |
| Linear SVC | 0.95 | 0.91 | 1.00 |
| SVC | 0.96 | 1.00 | 0.92 |
| Gaussian NB | 0.96 | 1.00 | 0.92 |
| Decision Tree Classifier | 0.74 | 0.82 | 0.67 |
| Quadratic Discriminant Analysis | 0.60 | 0.45 | 0.75 |
| KNeighbors Classifier | 0.88 | 1.00 | 0.75 |
| Gaussian Process Classifier | 0.83 | 0.91 | 0.75 |

**Table S3.** Important bacterial taxa selected by model classification analysis. The importance for each microorganism in the prediction of models is reported as mean and standard deviation (Std) values.

| **Feature** | **Score Mean** | **Score Std** | **Direction** |
| --- | --- | --- | --- |
| *Enterococcus* | 0.0154 | 0.0079 | COVID-19 |
| *Pseudomonas* | 0.0150 | 0.0076 | COVID-19 |
| *Streptococcus* | 0.0118 | 0.0076 | COVID-19 |
| *Capnocytophaga* | 0.0063 | 0.0043 | COVID-19 |
| *Tepidiphilus* | 0.0056 | 0.0045 | COVID-19 |
| *Porphyromonas* | 0.0044 | 0.0058 | COVID-19 |
| *Staphylococcus* | 0.0044 | 0.0046 | COVID-19 |
| *Veillonella* | 0.0023 | 0.0038 | COVID-19 |
| *Schlegelella* | 0.0428 | 0.0146 | CTRL |
| UCG-005 | 0.0128 | 0.0078 | CTRL |
| *Faecalibacterium* | 0.0085 | 0.0066 | CTRL |
| *Bifidobacterium* | 0.0053 | 0.0046 | CTRL |
